# Efficacy of hydrodistension for frozen shoulder: A systematic review and meta-analysis

**DOI:** 10.1097/MD.0000000000038388

**Published:** 2024-05-31

**Authors:** Tianpeng Chen, Wei Li, Yong Zhong, Tianle Chen, Xiaolin Shi

**Affiliations:** a Nanchang Hongdu Hospital of Traditional Chinese Medicine, Nanchang, China; b The Second School of Clinical Medicine, Zhejiang Chinese Medical University, Hangzhou, China; c The First School of Clinical Medicine, Gannan Medical University, Ganzhou, China; d The Second Affiliated Hospital of Zhejiang Chinese Medical University, Hangzhou, China.

**Keywords:** frozen shoulder, hydrodistension, meta-analysis, motor function, pain

## Abstract

**Background:**

The aim of this study was to provide comprehensive and reliable evidence for the treatment of pain and motor function in patients with frozen shoulder (FS) with hydrodistension

**Methods:**

The research including randomized controlled trials (RCTs) for FS that compared hydrodistension with routine treatments to controls was searched and screened in the PubMed, Library of congress, Cochrane Library, Web of Science, China National Knowledge Infrastructure (CNKI), Wanfang Data Knowledge Service Platform, China Science and Technology Journal Database. Constant-Murley score (CMS) for shoulder function and visual analog scale (VAS) for pain must be assessed. RevMan 5.3 software was used to evaluate the bias and quality of the included studies.

**Results:**

We found that analgesic effects (MD: −1.07; 95% CI: −1.94 to −0.20; *P* < .00001; *I*^2^ = 99%) and function (MD: 8.54; 95% CI: 3.35 to 13.71; *P* < .00001; *I*^2^ = 97%) were better in the groups where hydrodistension plus routine treatments were used to treat FS compared to other interventions.

**Conclusion:**

The result suggests that hydrodistension is of great clinical significance in alleviating pain and improving function to patients with FS.

## 1. Introduction

Frozen shoulder (FS) is a common shoulder disorder characterized by shoulder pain and a limited range of motion of the shoulder joint.^[1,2]^ The etiology of FS is unclear and may be related to factors such as Dupuytren contracture, inflammatory arthritis, thyroid disease, diabetes, heart disease, cervical spondylosis and chronic inflammation.^[3,4]^ FS is a very common condition that causes a great deal of morbidity. Lifting heavy objects and working with hands at or above the shoulder are independent risk factors for shoulder pain.^[5]^ An epidemiologic survey of FS in China showed that there were 192 cases of FS among 330 patients, with a prevalence rate of 58.18%, which was positively correlated with the age of the worker, the length of the working hours, and the busyness of the work.^[6]^ FS affects the quality of daily life and requires prompt treatment.

Current treatments for FS include drugs, local injections, physical therapy, water dilation, and surgery.^[7,8]^ Treatment of FS should be determined on an individual patient basis, such as severity and duration of symptoms. Conservative treatment is mostly adopted at the initial stage, and most patients can achieve satisfactory results after conservative treatment. Hydrodistension is a treatment that dilates the attached shoulder capsule by injecting large amounts of saline and small amounts of steroid hormones.^[9]^ It has been shown that water dilation is clinically effective in treating FS and can effectively relieve pain.^[10,11]^ However, there are a few meta-analyses evaluating the effectiveness of hydrodilatation in the treatment of FS. Therefore, in this study, we aimed to investigate the effects of hydrodistension on pain and function in FS.

## 2. Methods

It is not a clinical trial, so it does not require ethical approval.

### 2.1. Inclusion criteria

The inclusion criteria were as follows:

(1) Randomized controlled trials (RCTs);
(2) Patients who have been diagnosed with FS;
(3) The experimental groups were treated with hydrodistension plus routine treatments, the control groups were treated with routine treatments;
(4) There were no restrictions to the duration and severity of the disease;
(5) The languages were limited to English and Chinese;
(6) There were no restrictions on whether adopt a blind method or not;
(7) Constant-Murley score (CMS) for shoulder function and visual analog scale (VAS) for pain must be assessed.

### 2.2. Exclusion criteria

The exclusion criteria were as follows:

(1) Shoulder joint pain and dysfunction caused by diseases other than FS;
(2) The data were incomplete and could not be obtained by contacting the authors.

### 2.3. Search strategy

Seven databases, including PubMed, Library of Congress, Cochrane Library, Web of Science, China National Knowledge Infrastructure (CNKI), Wanfang Data Knowledge Service Platform and China Science and Technology Journal Database, were searched from inception to October 2023. Two authors (T.C. and T.C.) searched and screened all articles independently. A combination of MeSH terms and a free word search strategy was used. The search strategy was as follows:

1. Search: (((((frozen shoulder [MeSH Terms]) AND Shoulder Adhesive Capsulitis [MeSH Terms]) AND Adhesive Capsulitides, Shoulder [Title/Abstract]) AND Capsulitides, Shoulder Adhesive

[Title/Abstract]) AND Capsulitides, Adhesive [Title/Abstract]).

1. Search: ((((hydrodistension [MeSH Terms]) AND hydrotherapy [Title/Abstract]) AND hydrodistension [Title/Abstract]) AND Hydraulic release [Title/Abstract]) AND hydrotherapy [MeSH])
2. Search: randomized [Title/Abstract] OR (randomized controlled trial [Publication Type] OR placebo [Title/Abstract])

1 AND 2 AND 3

### 2.4. Data extraction and management

**Figure 1.**
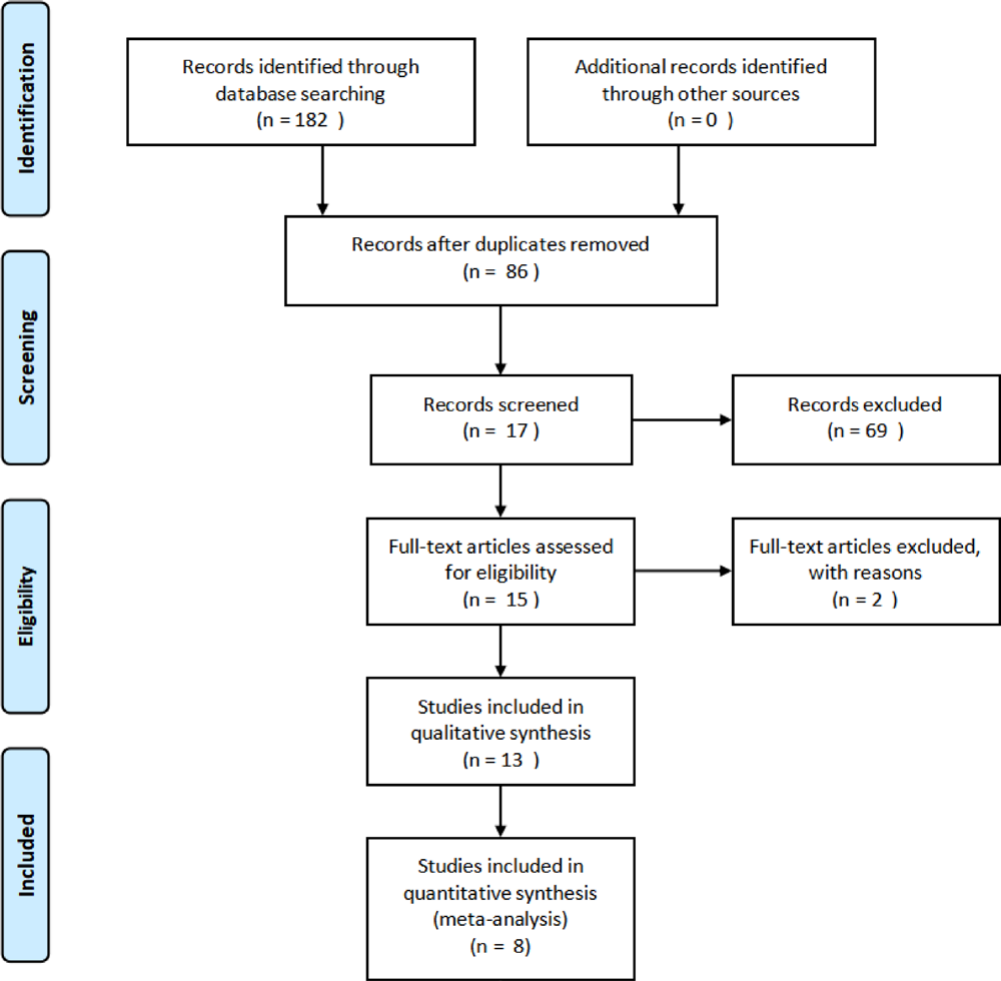
Selection process.

We used *NoteExpress V3* to extract articles from the above databases. Two authors (T.C. and T.C.) reviewed the titles and abstracts of all studies to determine whether to adopt them. If the 2 authors were not in agreement, another author (Y.Z.) reviewed and made the final decision. The PRISMA flow diagram illustrates the study selection process (Fig. 1).

We evaluated the bias assessment and quality classification of the included studies by using RevMan 5.3 (Nordic Cochrane Centre, The Cochrane Collaboration). Statistical Processing and Assessment of Heterogeneity For continuous variables, we extracted the mean difference (MD) and standardized MD (SMD) with 95% CI. Event ratios and sample sizes for dichotomous data were extracted. We used the *I^2^* statistic and the Cochran Q statistic with *P* values to evaluate heterogeneity. If significant heterogeneity was present (*I^2^* > 50% or *PQ* < .1), we used the random effects model; otherwise, a fixed-effects model was adopted.

## 3. Results

### 3.1. Study selection

There were 182 articles identified by searching electronic databases. Based on the inclusion and exclusion criteria, 8 RCTs were finally included.^[12–19]^ We read the included studies to record the characteristics of the studies, including gender, age, disease duration, and treatment. The characteristics of the studies are shown in Table 1.

**Table 1:** The characteristics of studies.

Studies		Case number	Age (yr)	Sex (male/female)	Course of disease (month)	Treatment mode
Liu WD 2013^[12]^	e.g.	50	48.32 +0.45	21/29	10.66 +0.63	hydrodistension + needle knife
	CG	50	48.74 +0.41	23/27	11.29 +0.52	Steroid injection + needle knife
Elnady B 2020^[13]^	e.g.	30	47.6 +13.5	9/21	8.31 +2.68	hydrodistension + Steroid injection
	CG	30	45.4 +4.9	8/22	9.1 +2.93	Steroid injection
Kang SQ 2020^[14]^	e.g.	33	55.9 +7.7	10/23		hydrodistension + needle knife
	CG	33	54.4 +7.9	11/22		Manual release of shoulder joint
Wang YH 2020^[15]^	e.g.	30	58.54 +14.37	12/18	6.4 + 0.31	hydrodistension
	CG	30	58.19 +14.51	11/19	6.5 + 0.25	Shoulder arthroscope
Wang W 2021^[16]^	e.g.	29	54.34 +4.42	14/15	6.76 + 2.81	Hydrodistension + Manual release of shoulder joint
	CG	29	56.38 +6.28	12/17	6.59 + 2.60	Steroid injection + Manual release of shoulder joint
Dimitri-Pinheiro S 2023^[17]^	e.g.	50	56.78 +3.21	23/27	5.64 + 1.32	hydrodistension
	CG	50	57.32 +4.05	26/24	6.21 + 2.10	Steroid injection
DingyuanLuo 2023^[18]^	e.g.	55	53.4 + 4.61	30/25	5.18 + 2.12	Hydrodistension + Steroid injection
	CG	55	54.82 + 3.44	26/29	5.07 + 1.64	Steroid injection
Zhu Y 2023^[19]^	e.g.	40	55.20 + 3.3	13/22		Hydrodistension + Manual release of shoulder joint
	CG	40	64.1 + 3.5	14/26		Manual release of shoulder joint

### 3.2. Methodological quality and the risk of bias

**Figure 2.**
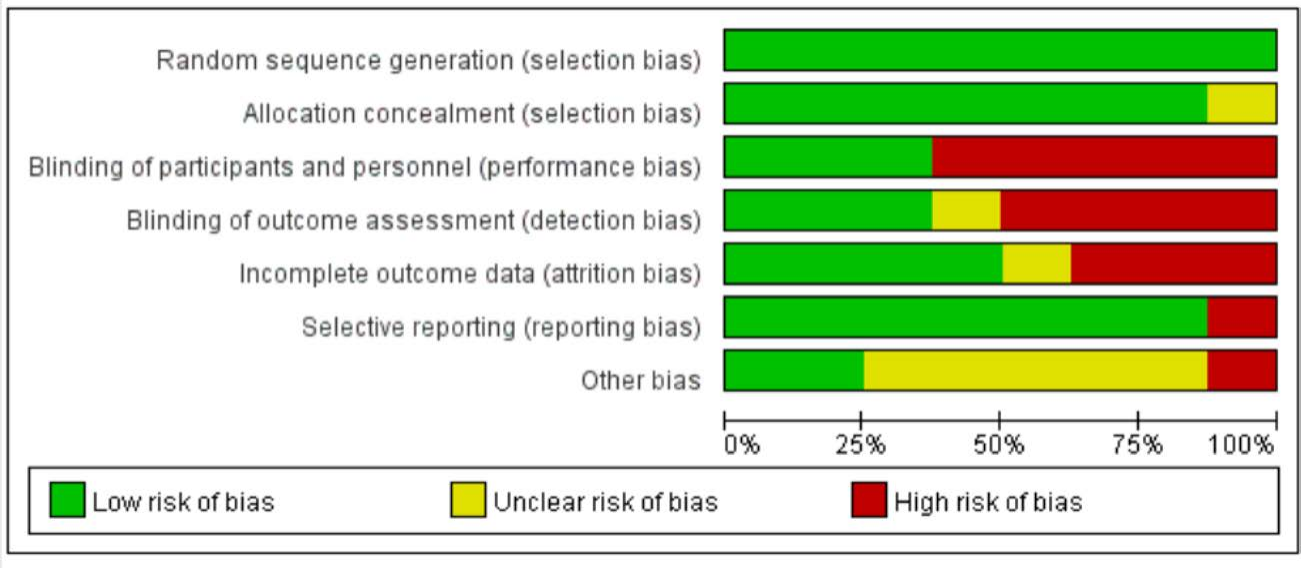
Risk of bias summary.

**Figure 3.**
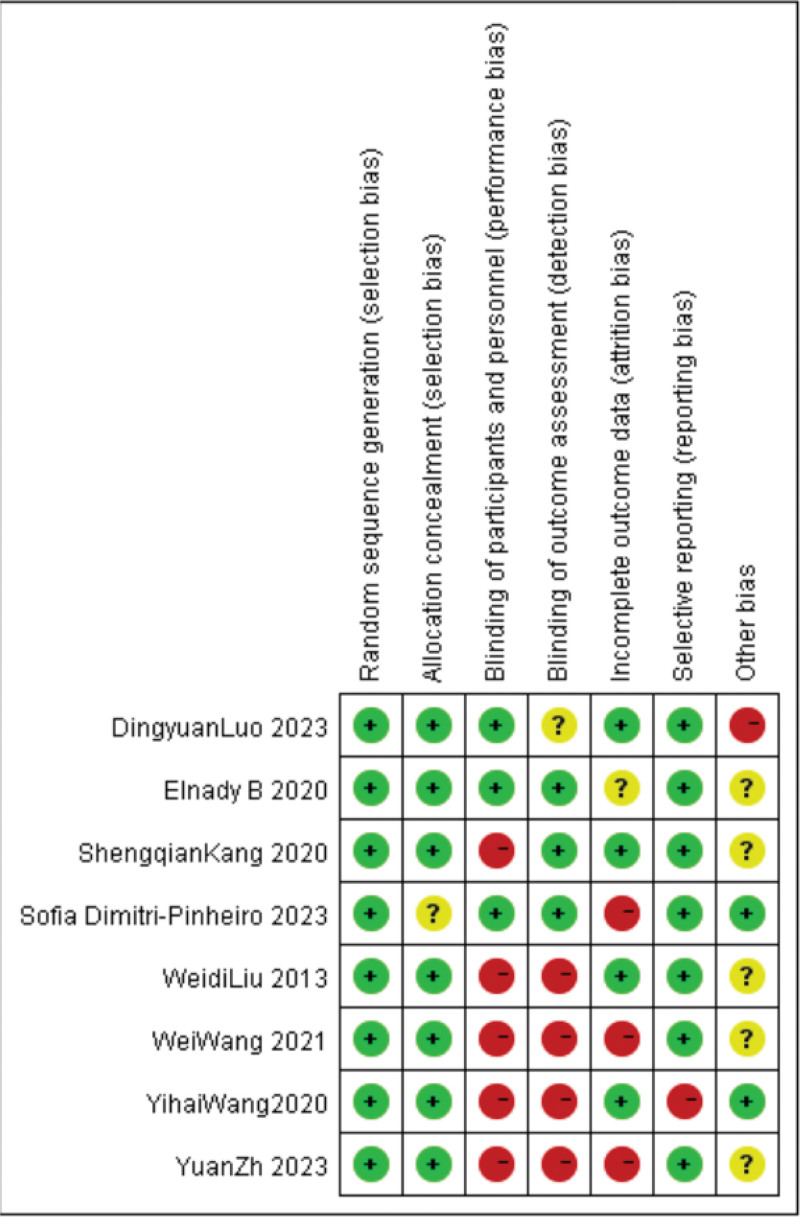
Assessment of risk of bias in all included randomized controlled trials.

The risk of bias table and summary are shown in Figures 2 and 3. Among the included studies, we used appropriate methods to minimize bias and were considered to have a low risk of selection bias, performance bias, detection bias, attrition bias, reporting bias, and other bias. Among the 8 included studies, 2 were of high quality with a low risk of bias, while the other 6 had varying degrees of risk of bias.

### 3.3. Meta-analysis

#### 3.3.1. VAS

**Figure 4.**
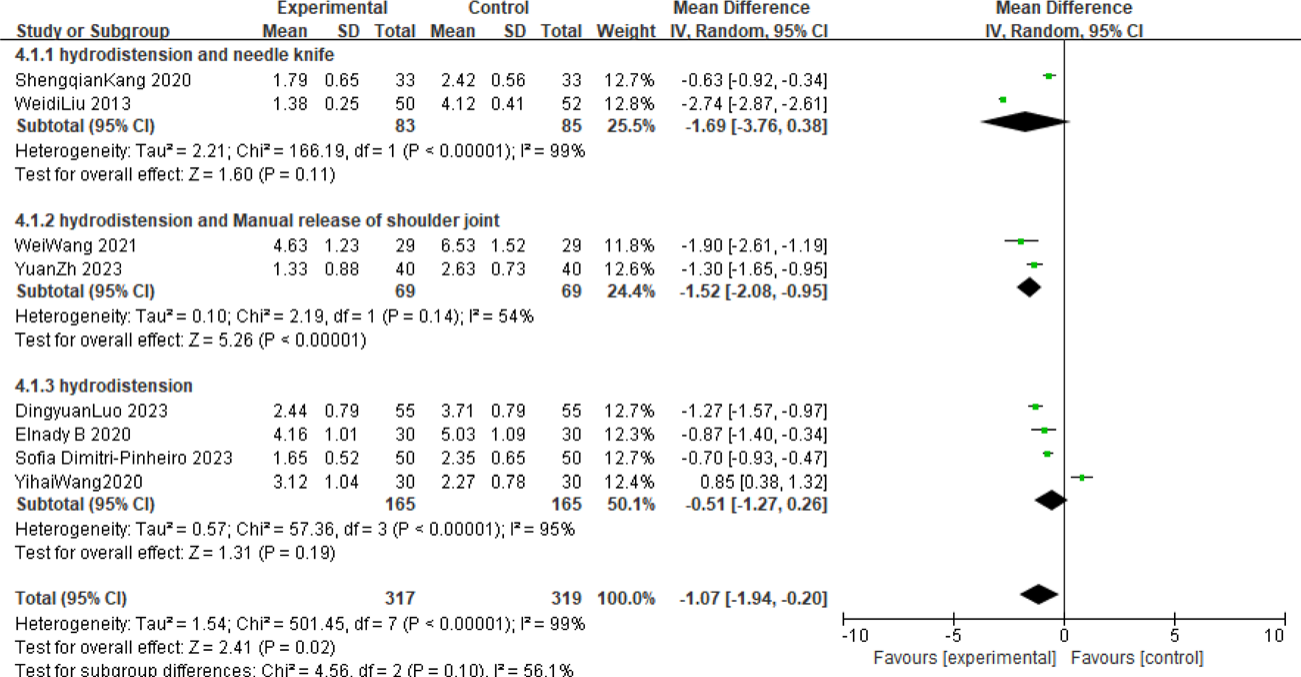
Forest plot of the comparison of visual analog scale (VAS) between hydrodistension combined with routine treatments and routine treatments.

Meta-analysis showed that hydrodistension combined with conventional treatment was more effective in reducing pain intensity than conventional treatment (MD: −2.13; 95% CI: −1.21 to −2.05; *P* < .00001, *I*^2^ = 99%). The results showed that hydrodistension had an advantage over conventional treatment in patients with FS. Due to the high heterogeneity, we performed a subgroup analysis based on treatment style, but the results were not satisfactory, and the overall heterogeneity remained high except for a significant reduction in heterogeneity in 1 group (MD: −1.07; 95% CI: −1.94 to −0.20; *P* < .00001, *I^2^* = 99%). The result is shown in Figure 4.

#### 3.3.2. CMS

**Figure 5.**
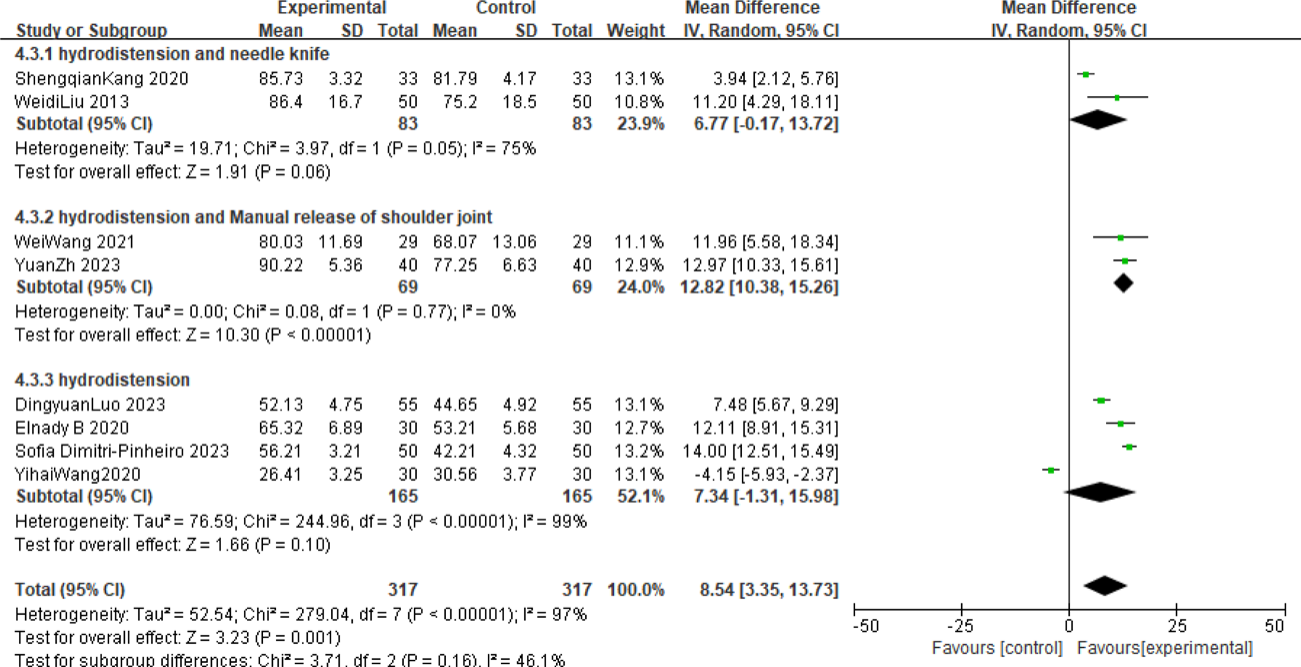
Forest plot of the comparison of Constant-Murley score (CMS) between hydrodistension combine with routine treatments and routine treatments.

The effect of hydrodistension combined with conventional treatment on shoulder joint function was evaluated in 8 trials. Meta-analysis showed that hydrodistension combined with conventional treatment was more effective in CMS, as shown in Figure 5 (MD: 8.54; 95% CI: 3.35 to 13.71; *P* < .00001, *I^2^* = 97%). However, heterogeneity was high. We used a random effects model, reference deletion method and subgroup analysis to reduce heterogeneity, but the results were not satisfactory.

## 4. Discussion

Although the pathogenesis of FS is still unclear, the pain and limitations of shoulder joint activities seriously affect the patients’ work and daily lives. The shoulder joint not only has a large range of motion but is also frequently used, and many activities in daily life cannot be separated from the shoulder joint, which accelerates the degeneration of the soft tissues around the joint, and is prone to aseptic inflammation, resulting in the formation of FS. The optimal timing of surgical intervention for FS patients remains controversial. On the one hand, FS is considered to be a self-limiting disease and the symptoms will subside with time. On the other hand, surgical treatment may have certain complications, such as infection and nerve damage, which should be considered when choosing surgical treatment. If FS is not effectively treated, it will seriously affect the functional activities of the shoulder joint, and in severe cases, symptoms such as muscle atrophy may occur. Most patients will choose conservative treatment at the initial stage, and hydrodistension is a good choice. Hydrodilatation is performed by inserting a syringe into the glenohumeral joint and injecting a contrast agent to observe whether the rotator cuff is ruptured or not. A decrease in the volume of the joint cavity can further support the diagnosis of FS, and then saline is slowly injected to expand the joint capsule through the pressure of the liquid until a sudden decrease in the pressure of the injection is felt, which indicates that the joint capsule is ruptured, which is the key to the therapeutic effect, and lastly, steroids can be injected into the joint cavity as well.^[20]^ Finally, functional exercises are also required immediately after surgery. Lädermann published a meta-analysis comparing physical therapy, intra-articular hormone injections, and hydrodilatation, and their results showed that hydrodilatation demonstrated more significant efficacy than the other 2 treatment modalities, resulting in better pain control and shoulder function.^[21]^ However, there are fewer studies on the treatment of FS with hydrodistension, and more clinical studies are needed to prove the clinical efficacy of hydrodistension. For patients who are not well treated with conservative treatment, further surgical treatment is still needed.

This meta-analysis compared the effectiveness of hydrodilatation combined with conventional treatment in FS. We found that the use of hydrodilatation combined with conventional treatment provided better analgesic effect (MD: −1.07; 95% CI: −1.94 to −0.20; *P* < .00001; *I*^2^ = 99%) and improved mobility function (MD: 8.54; 95% CI: 3.35 to 13.71; *P* < .00001; *I*^2^ = 97%) in FS compared with other treatment measures. However, this meta-analysis has some limitations: Firstly, the number of included studies is small and the evidence may not be strong. Secondly, the meta-analysis was highly heterogeneous, which might be related to the small number of studies and the long years of study. Therefore, more RCTs studies are needed to provide more favorable evidence.

## 5. Conclusion

The results of this meta-analysis suggest that hydrodistension has an important clinical effect on relieving pain and improving function in patients with FS. However, further rigorously designed trials and more relevant studies are needed to confirm the conclusion.

## Author contributions

**Conceptualization:** Tianpeng Chen, Wei Li.

**Data curation:** Tianpeng Chen, Tianle Chen.

**Formal analysis:** Yong Zhong.

**Methodology:** Yong Zhong, Xiaolin Shi.

**Project administration:** Yong Zhong, Xiaolin Shi.

**Software:** Yong Zhong.

**Writing – original draft:** Tianpeng Chen.

**Writing – review & editing:** Wei Li.
